# IUC24409-84 Evaluation of a transurethral laser ablation (TULA) service for superficial bladder cancer management in an outpatient setting in a UK district general hospital

**DOI:** 10.1093/oncolo/oyaf248.019

**Published:** 2025-08-29

**Authors:** P Carter, M Akram, R Ritchie

**Affiliations:** Salisbury NHS Foundation Trust, United Kingdom; Salisbury NHS Foundation Trust, United Kingdom; Salisbury NHS Foundation Trust, United Kingdom

## Abstract

**Background:**

While transurethral resection of bladder tumour (TURBT) remains the standard treatment for non-muscle invasive bladder cancer (NMIBC), complication rates in the literature vary widely from 5.1% to over 26%. Transurethral laser ablation (TULA) under local anesthetic in the outpatient setting is a safe and minimally invasive option for the treatment of non-muscle invasive bladder cancer, particularly for frail and co-morbid patients who may be unsuitable for a general anesthetic. This review aims to evaluate the safety and efficacy of an outpatient TULA service in a UK district general hospital.

**Methods:**

A retrospective review was conducted of 50 consecutive patients who underwent TULA in 2023. Data were collected from the electronic patient records, including basic demographics, clinical documentation, tumor histology at diagnosis, TULA indication, post-procedure complications, and recurrence rates up to 2 years.

**Results:**

The mean patient age was 80.66 (range 49-98); 32 were male and 18 were female. Indications for TULA included possible recurrence (68%), red patch (18%), new tumor in a highly co-morbid patient (12%), and field change (2%). Most patients (60%) had low-grade G2 pTa as their original histological diagnosis. Post-procedure urinary tract infection (UTI) rate at 2 weeks was 2%, and hematuria at one month was also 2%. No cases of urinary retention, hydronephrosis on upper tract imaging at 6 months, bladder perforation or post-procedure admission to hospital were recorded. Bladder cancer recurrence rates at 6 months, 1 year, and 2 years were 28.3%, 42.9%, and 54.1%, respectively. During the 2-year follow-up period, 40% of patients went on to have further TULA. 14% of patients died within the 2 years, with only one death attributed to metastatic bladder cancer.

**Conclusions:**

Outpatient TULA is a safe and well-tolerated treatment option for selected patients with non-muscle invasive bladder cancer, and our data shows comparable recurrence rates to other studies. It offers an effective alternative to a general anesthetic procedure and negates the need for stopping anticoagulation in higher-risk patients. It has a low complication rate, making it safe, especially for frail, co-morbid patients who may not be fit for general anesthetic procedures.

